# EphA4 loss improves social memory performance and alters dendritic spine morphology without changes in amyloid pathology in a mouse model of Alzheimer’s disease

**DOI:** 10.1186/s13195-019-0554-4

**Published:** 2019-12-12

**Authors:** Lindsay Poppe, Laura Rué, Mieke Timmers, Annette Lenaerts, Annet Storm, Zsuzsanna Callaerts-Vegh, Gilles Courtand, Antina de Boer, Silke Smolders, Philip Van Damme, Ludo Van Den Bosch, Rudi D’Hooge, Bart De Strooper, Wim Robberecht, Robin Lemmens

**Affiliations:** 1 Department of Neurosciences, Experimental Neurology, and Leuven Brain Institute (LBI), KU Leuven – University of Leuven, Leuven, Belgium; 2 Laboratory of Neurobiology, Center for Brain and Disease Research, VIB, Leuven, Belgium; 3 Laboratory of Biological Psychology, Faculty of Psychology and Educational Sciences, KU Leuven - University of Leuven, Leuven, Belgium; 4 mINT Animal Behavior Core Facility, Faculty of Psychology, KU Leuven, Leuven, Belgium; 5 Institut de Neurosciences Cognitives et Intégratives d’Aquitaine, Unité Mixte de Recherche 5287, Centre National de la Recherche Scientifique, Université de Bordeaux, 33076 Bordeaux, France; 6 Department of Neurology, University Hospitals Leuven, Herestraat 49, B-3000 Leuven, Belgium; 7 VIB Center for Brain and Disease Research, Leuven, Belgium; 8 Department of Neurosciences, Katholieke Universiteit Leuven, Leuven, Belgium; 9 UK Dementia Research Institute at University College London, London, UK

**Keywords:** EphA4, Ephrins, Alzheimer’s disease, Dendritic spine, Synapse, Social memory, APPPS1

## Abstract

**Background:**

EphA4 is a receptor of the ephrin system regulating spine morphology and plasticity in the brain. These processes are pivotal in the pathophysiology of Alzheimer’s disease (AD), characterized by synapse dysfunction and loss, and the progressive loss of memory and other cognitive functions. Reduced EphA4 signaling has been shown to rescue beta-amyloid-induced dendritic spine loss and long-term potentiation (LTP) deficits in cultured hippocampal slices and primary hippocampal cultures. In this study, we investigated whether EphA4 ablation might preserve synapse function and ameliorate cognitive performance in the APPPS1 transgenic mouse model of AD.

**Methods:**

A postnatal genetic ablation of EphA4 in the forebrain was established in the APPPS1 mouse model of AD, followed by a battery of cognitive tests at 9 months of age to investigate cognitive function upon EphA4 loss. A Golgi-Cox staining was used to explore alterations in dendritic spine density and morphology in the CA1 region of the hippocampus.

**Results:**

Upon EphA4 loss in APPPS1 mice, we observed improved social memory in the preference for social novelty test without affecting other cognitive functions. Dendritic spine analysis revealed altered synapse morphology as characterized by increased dendritic spine length and head width. These modifications were independent of hippocampal plaque load and beta-amyloid peptide levels since these were similar in mice with normal versus reduced levels of EphA4.

**Conclusion:**

Loss of EphA4 improved social memory in a mouse model of Alzheimer’s disease in association with alterations in spine morphology.

## Background

Alzheimer’s disease (AD) is a devastating neurodegenerative disorder, characterized by progressive decline in memory, together with multiple cognitive impairments compromising the patient’s daily life [1]. Pathological features observed in AD brains are the extracellular amyloid depositions of beta-amyloid (Aβ) peptides into senile plaques and the intraneuronal neurofibrillary tangles, composed of hyperphosphorylated tau protein [2]. Moreover, neuroinflammatory features, such as astrogliosis and microgliosis, are present in AD patients and experimental animal models [3]. Dysfunction and loss of synapses are early events in AD and correlate with cognitive impairments in patients suggesting synaptic dysfunction as the underlying cause of cognitive deficits in patients [4–8]. AD is the underlying cause of 60–70% of the dementia cases. It is estimated that 40 million people worldwide are affected by the disease, and by the year 2050, this number will have been tripled [9], creating a massive socioeconomic burden. So far, no cure for AD exists and current treatments generally concentrate on multidisciplinary care. Meanwhile, research mainly focuses on targeting the amyloid and tau pathology present in the brains of AD patients [10–12]. However, therapies aimed at synapse dysfunction are valid alternative strategies [13].

EphA4 is a tyrosine kinase receptor of the ephrin system which is highly expressed in the nervous system [14]. During development of the nervous system, EphA4 functions as an important repellant cue in axon guidance [15], whereas in adults, hippocampal EphA4 is a crucial mediator of synapse morphology, synaptic functionality, and plasticity. Interaction of EphA4 with ephrin-A3 in hippocampal slices induces spine retraction, a process involved in synapse pruning [15–17]. EphA4 modulates the excitability of the synapse via regulation of the local levels of AMPA receptors and glial glutamate transporters during physiological processes such as homeostatic plasticity and long-term potentiation [18, 19]. Therefore, EphA4 has become an interesting target for disorders characterized by synaptic dysfunction, such as depression or AD. In mice with an induced depressive-like phenotype, inhibition of EphA4 ameliorates spine loss [20]. In the context of AD, genetic reduction or pharmacological blockage of EphA4 rescues Aβ-induced dendritic spine loss and long-term potentiation (LTP) deficits in cultured hippocampal slices and primary hippocampal cultures [21, 22]. In addition, reversal of Aβ-dependent memory impairment in a sortilin-related receptor with LDLR class A repeats (SORLA)-overexpressing mouse is suggested to be associated with decreased EphA4 activation and redistribution to the postsynaptic densities [23]. In this study, we investigated whether the beneficial effects of reduced EphA4 signaling on Aβ-induced spine pathology could be translated into a mouse model of AD and ultimately ameliorate cognitive performance. We show that EphA4 loss increased social memory performance in APPPS1 mice, together with increases in spine length and spine head width.

## Materials and methods

### Animal origin, housing, breeding, and study approval

We used a previously generated transgenic mouse model of AD (human amyloid precursor protein {hAPP} KM670/671NL; human presenilin 1 {hPS1} L166P, further on referred to as APPPS1 mice), which develops Aβ pathology early in life accompanied by astro- and microgliosis and cognitive decline from 9 months on [24]. We crossbred these mice with EphA4^flox/flox^ (EphA4^tm1.1Bzh^/J; stock number: 012916, The Jackson Laboratory) [25] and Camk2aCre (B6.Cg-Tg(Camk2a-Cre)T29-1Stl/J; stock number: 005359, The Jackson Laboratory) [26] mice to generate APPPS1 mice with a profound loss of EphA4 in the forebrain from the first postnatal weeks on. All mice were maintained in a C57/Bl6J background. All experiments were performed with mixed cohorts containing similar numbers of male and female mice.

Mice were housed in the “KU Leuven” animal facilities with a 12-h light-dark cycle at a temperature of 20 °C. Animals were given free access to standard rodent chow and water. All animal experiments were carried out in accordance with the National Institutes of Health guide for the care and use of laboratory animals (NIH publications No. 8023, revised 1978). Experiments were designed to minimize animal discomfort and were approved by the Ethical Committee for Animal Research of the University of Leuven, Belgium (P178/2013).

### Tissue collection

After cognitive assessment, all mice were anesthetized with 10% Nembutal (Ceva chemicals). For immunoblot and Aβ extractions, mice were transcardially perfused with phosphate-buffered saline (PBS) and the brain was microdissected to collect hippocampi and cortices. Samples were snap frozen in liquid nitrogen and stored at − 80 °C until further analysis. For RNA in situ hybridization, amyloid plaque analysis, and Golgi-Cox staining, mice were transcardially perfused with PBS and 4% paraformaldehyde (PFA). For RNA in situ hybridization and amyloid plaque analysis, brains were further fixated by overnight incubation in 4% PFA and cryoprotected in subsequently 10%, 20%, and 30% sucrose gradients. Brains were frozen in ice cold isopentane and stored at − 80 °C until further analysis. For Golgi-Cox staining, brains were processed as described below.

### Immunoblot

Mouse hippocampi and cortices were homogenized in T-PER® Tissue protein extraction reagent (Thermo Scientific, 78510) with protease (cOmplete; Roche, 11697498001) and phosphatase (phosphostop; Roche, 4906845001) inhibitors using the MagNaLyser (Roche). Protein concentration was determined with the Pierce BCA protein assay kit (Thermo Scientific, 23225). For electrophoresis, we used 4–20% precast acrylamide gels (Mini-PROTEAN® TGX™; Bio-Rad, cat#456-1096), and 15 or 20 μg of protein were loaded for each sample. Proteins were transferred to Immobilon-P (PVDF) membrane (Millipore, IPVH00010) and subsequently blocked with 5% nonfat-dry milk (Blotting-Grade Blocker; Bio-Rad, cat#170-6404) and 5% bovine serum albumin (Serva Electrophoresis GmbH, 1193003) in Tris-buffered saline with 0.001% Tween® (TBS-T) for 1 h at room temperature. Membranes were incubated with the following primary antibodies: C-terminal mouse anti-EphA4 (1/500; Invitrogen, 37-1600), mouse anti-GAPDH (1/10000; Thermo Scientific, AM4300), rat anti-PS1 (1/500; Millipore, MAB1563), and an in-house made rabbit anti-APP (B63, 1/5000) antibody. Anti-mouse-HRP, anti-rat-HRP, and anti-rabbit-HRP (all 1/5000, DAKO) were used as secondary antibodies. ECL or FEMTO ECL (Thermo Scientific, 32106 and 34095) was used as a substrate, and the signal was detected using LAS4000 (GE Healthcare). Band optical density was quantified with the ImageQuantTL software (EG Biosciences).

### RNA in situ hybridization

Cryosections of 30-μm thickness were post-fixated, dehydrated, and dried. In situ hybridization was performed using the commercially available RNAscope® Multiplex Fluorescent Reagent Kit v2 (Advanced Cell Diagnostics), as stated in the manufacturer’s instructions. Slides were incubated overnight with a probe specific for EphA4 (Mm-EphA4-C1, ACD Diagnostics), and the signal was amplified using TSA Plus Cyanine 3 (1/500, Perkin Elmer). Cell nuclei were stained with Hoechst, and slides were mounted using Prolong® Gold antifade mountant (Thermo scientific, P36934). Image z-stacks were taken every 2 μm for a total depth of 8 μm with a Leica TSC SP8 confocal laser scanning microscope (Leica Microsystems Heidelberg GmbH) with an HC PL APO CS2 20x/0.75 dry lens and a pinhole of 0.6 Airy Units.

### Behavioral testing

#### Open field

Exploration and anxiety were studied in the open field exploration test. Mice were dark-adapted for 30 min before being placed in the open field arena (50 × 50 cm^2^). After 1 min of habituation in the arena, exploratory behavior was recorded for 10 min using Anymaze software (Stoeltus) and total distance was measured as a parameter for locomotor activity. As mice will typically spend more time in the “protected” periphery of the arena, and increased exploration of the “unprotected” center of the field demonstrates anxiolytic behavior, the time spent in the open field center and in the small periphery were also measured.

#### Morris water maze

Morris water maze was performed to study spatial learning and memory capacity. The standard hidden-platform acquisition of the Morris water maze was used [27, 28]. The maze consisted of a large circular pool (diameter 150 cm) filled with water (26 °C) to a depth of 16 cm. Water was made opaque with non-toxic white paint to prevent animals from seeing the platform. The pool was divided in four imaginary quadrants, and a circular platform (diameter 15 cm) was hidden 1 cm beneath the water surface at a fixed position. The pool was localized at the center of a room with various fixed cues (e.g., posters, computers, tables). The experimenter always sat in the same place. Mice were trained for 10 days to find the hidden platform during four trials per training day with a trial interval of 15 min. Mice were placed in the pool in one of the four quadrants, and the starting quadrant was alternated during a training day. When mice were not able to find the platform within 100 s, they were guided to the platform and had to stay on it for 10 s, before being returned to their cages. Escape latency (average duration to find the platform during the four trials per day) was recorded with Ethovision software (Noldus). After the fifth and tenth learning day, mice had 2 days of rest followed by a probe trial to evaluate spatial retention memory. During this first and second probe trial, the platform was removed, and the time spent in each quadrant was measured for 100 s.

#### SPSN

The sociability/preference for social novelty test (SPSN test) was performed in a large transparent Plexiglas box divided into three compartments by transparent Plexiglas walls with small square openings as described previously [29]. Briefly, a holding cage was placed in the middle of the two outer-most compartments and the procedure consisted of three consecutive steps. First, mice were acclimatized in the middle compartment for 5 min (acclimatization phase). In a second phase (sociability trial), an unfamiliar mouse of the same sex (novel mouse) was introduced in one of the holding cages in one outer compartment, while the other holding cage remained empty. Exploratory behavior towards the novel mouse and the empty holding cage was measured for 10 min. In the third phase (social memory trial), another unfamiliar mouse of the same sex (novel mouse) was introduced in the other compartment. During this last phase, exploratory behavior towards the familiar and the novel mouse was recorded for 10 min. Exploratory behavior was defined as sniffing time towards a holding cage (with or without a mouse in it). The location of the novel and familiar mouse was counterbalanced across testing animals, and the apparatus was cleaned thoroughly with water after each mouse and with ethanol when a mouse of a different gender was tested. Behavior was recorded using Anymaze software (Stoeltus), and sniffing times (ST) were measured manually by watching the video recordings. We calculated preference ratio during the sociability trial as ST novel mouse/(ST novel mouse + ST empty cage) and recognition ratio during the social memory trial as ST novel mouse/(ST familiar mouse + ST novel mouse).

### Golgi-Cox staining and spine analysis

Brains were stained using the FD Rapid GolgiStain kit (FD NeuroTechnologies, PK401) according to the manufacturer’s instruction. In brief, brains were immersed in a 1:1 mixture of FD Solution A and B for 2 weeks at room temperature in the dark. Next, brains were transferred to FD Solution C for 48 h at 4 °C in the dark. After the first 24 h, Solution C was renewed. Brains were frozen and kept at − 80 °C until further processing. Coronal cryosections of 100-μm thickness were cut with a CryoStar NX70 cryostat (Thermo Fischer Scientific). Slices were transferred to small droplets of FD Solution C on gelatin-coated slides (FD NeuroTechnologies, P0101). Sections were dried for at least 3 h at room temperature before staining. Further staining was performed as described in the product manual. For dendritic spine analysis, images of apical dendrites from ventral CA1 pyramidal neurons of the hippocampus were taken using a Leica TSC SP8 confocal laser scanning microscope (Leica Microsystems Heidelberg GmbH) with a HC PL APO CS2 63x/1.40 oil lens. A transmitted light detector was used to mimic bright-field imaging. Image z-stacks were obtained every 0.2 μm with a 2048 × 2048 pixel resolution. Dendritic segments of approximately 20 μm in length from two regions of the CA1 stratum radiatum (SR) were imaged: 30–120 μm from the soma (proximal SR) and 120–300 μm from the soma (distal SR). Minimum Z-projections were created and were loaded into the Neurolucida 360 software to trace dendritic segments and quantify dendritic spine numbers, length, and head thickness. Approximately six segments (of which not more than two segments from the same neuron) per region per mouse were included in the study.

### Amyloid plaque load

Mouse brains were cut in free-floating series of 30-mm-thick coronal sections using a CryoStar NX70 Cryostat (ThermoFisher Scientific). Every ninth section was assigned to one series. Consequently, every series is representative for the whole selected brain area. Sections were stored in PBS with 0.02% sodium azide at 4 °C. Slices were immersed in 0.015% Thioflavin S solution for 10 min and washed in PBS-T before incubation with TO-PRO®-3 staining solution (Thermo Scientific, T3605) for 30 min. Slices were mounted with Prolong® Gold antifade mountant (Thermo Scientific, P36934). Fluorescent images of one series per animal were made with a Leica DMI 6000B inverted microscope. Images covering the whole hippocampus in one section were made using a × 10 objective, and all these images were merged into one mosaic picture. An average of 13 mosaic pictures was made per animal, depending on the rosto-caudal extension of the hippocampus. Plaque number and plaque load (% of hippocampal area positive for Thioflavin S) were quantified using ImageJ software by Wayne Rasband (National Institutes of Health). In brief, particle analyzer was used and a threshold was set to only detect plaques. Particles larger than 75 μm^2^ were considered as plaques. The average of plaque densities and plaque burden (the percentage of hippocampal area covered with Thioflavin-S-positive amyloid plaques) for all pictures per animal was considered as representative for the whole hippocampus.

### Aβ extraction and ELISA

Mouse hippocampi were homogenized in T-PER® tissue extraction reagent (Pierce) supplemented with protease (cOmplete; Roche, Vilvoorde, Belgium) and phosphatase (phosphostop; Roche, Vilvoorde, Belgium) inhibitors using the MagNaLyser (Roche, Vilvoorde, Belgium). Homogenized hippocampi were centrifuged for 1 h at 4 °C at 100,000*g*, and supernatant was used for ELISA to measure TBS (tris-buffered saline) –soluble Aβ levels. For the GuHCl-soluble Aβ levels, pellets were dissolved via sonication in a 6 M GuHCl extraction buffer and centrifuged for 20 min at 4 °C at 130,000*g*. Supernatant was diluted 1/12 to reduce the concentration of GuHCl and was used for ELISA. Aβ40 and Aβ42 levels were determined using commercially available ELISA kits from Wako Chemicals (290-62601 and 294-64701).

### Statistical analysis

Unpaired two-tailed Student’s *t* test was used for the comparison of two means. One-way, two-way, and two-way with repeated measures ANOVA tests were used for multiple group analysis. Data were tested for normality using D’Agostino and Pearson’s or, in case of small sample sizes, the KS normality test. Kruskal-Wallis and Mann-Whitney *U* tests were used when the data was not normally distributed. Student’s *t* tests, one-way and two-way ANOVA, and Kruskal-Wallis and Mann-Whitney *U* tests were performed using GraphPad Prism software version 7 (GraphPad software Inc), while two-way with repeated measures ANOVA tests were performed with IBM SPSS Statistics 25 software (IBM). *p ≤ 0.05, **p ≤ 0.01, ***p ≤ 0.001, ****p ≤ 0.0001, ^+^*p* ≤ 0.05, ^++^*p* ≤ 0.01, ^++++^*p* ≤ 0.0001. If no asterisk or plus sign is shown in the graph, this implies no significance. All data represents means ± SEM.

## Results

### Generation of APPPS1 mice with loss of EphA4 protein in the forebrain

**Fig. 1:**
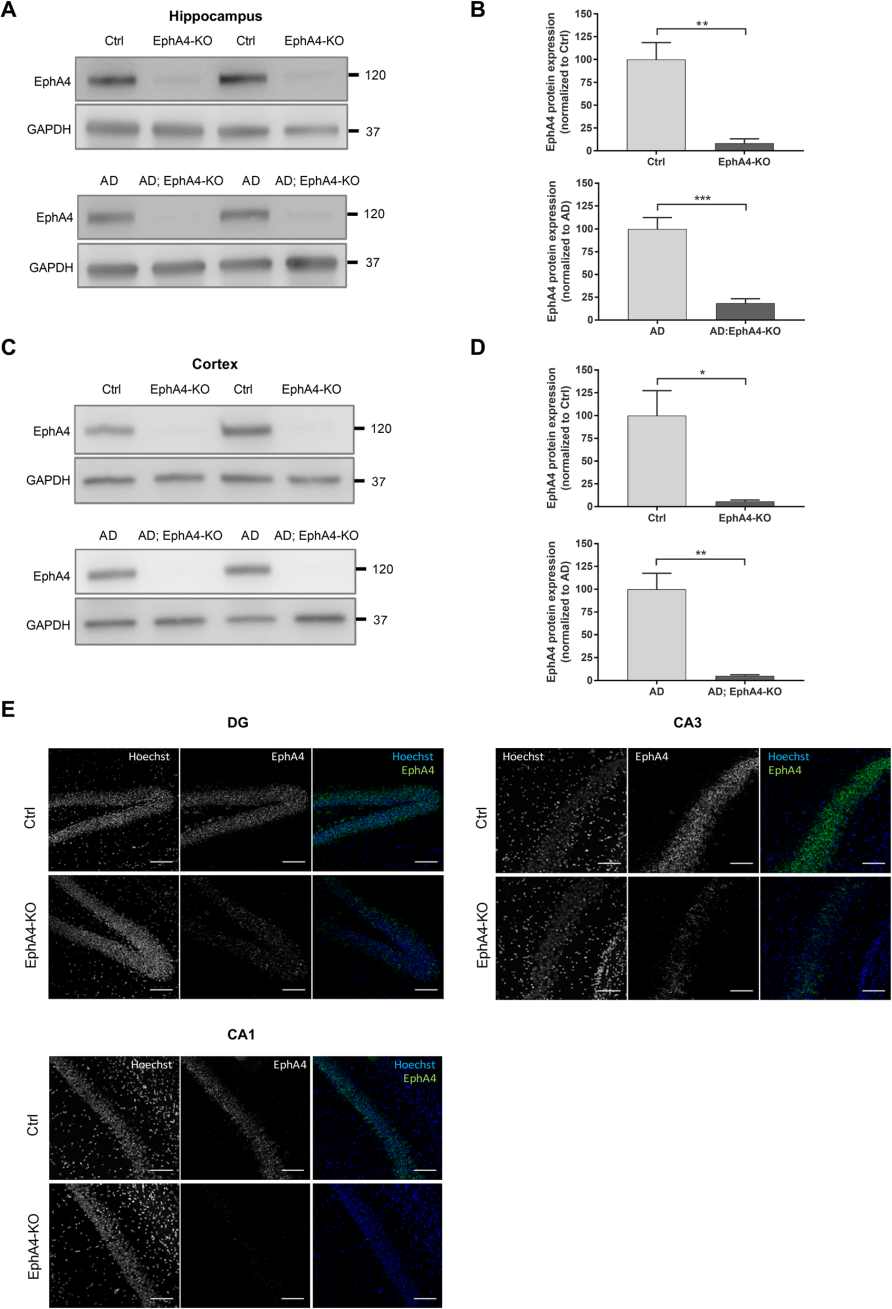
Reduction of EphA4 protein and mRNA levels in the hippocampus and cortex of APPPS1 mice. Representative images (**a**) and quantification (**b**) of a Western blot analysis of hippocampal EphA4 protein levels in Ctrl and EphA4-KO mice (upper blot) and AD and AD;EphA4-KO mice (lower blot) with GAPDH protein levels as a loading control (unpaired *t* test and Mann-Whitney test respectively, *n* = 4–8 mice/group). Representative images (**c**) and quantification (**d**) of a Western blot analysis of cortical EphA4 protein levels in Ctrl and EphA4-KO mice (upper blot) and AD and AD;EphA4-KO mice (lower blot). GAPDH protein levels were assessed to control for equal loading (unpaired *t* test, *n* = 4 mice/group). **e** Representative images of RNA scope with specific probes for EphA4 in the DG, CA3, and CA1 regions of the hippocampus of Ctrl and EphA4-KO mice. Hoechst was used to stain cell nuclei. *p ≤ 0.05, **p ≤ 0.01. Scale bar = 100 μm. Abbreviation: Ctrl control

In order to investigate the effect of EphA4 loss on memory function in a mouse model of AD, we crossbred APPPS1 mice with EphA4^flox/flox^ and Camk2aCre mice to specifically decrease EphA4 expression in the forebrain of APPPS1 mice. Western blotting confirmed a strong reduction in cortical and hippocampal EphA4 protein in EphA4^flox/flox^ x Camk2aCre (EphA4-KO) versus EphA4^flox/flox^ (Ctrl) mice and EphA4^flox/flox^ x Camk2aCre x APPPS1 (AD;EphA4-KO) versus EphA4^flox/flox^ x APPPS1 (AD) mice (Fig. 1a–d). To investigate the regional recombination efficiency in the hippocampus of the Camk2aCre mouse, we performed in situ hybridization using RNA scope with specific probes for EphA4. EphA4 mRNA levels were low in the dentate gyrus (DG) and CA3 regions of the hippocampus and almost absent in the CA1 region (Fig. 1e). In AD;EphA4-KO mice, hAPP and hPS1 expression was similar compared to AD mice, as determined by Western blot (Additional file 1: Figure S1 A-D).

### EphA4 knock-down improves social memory, but not spatial memory in APPPS1 mice

**Fig. 2:**
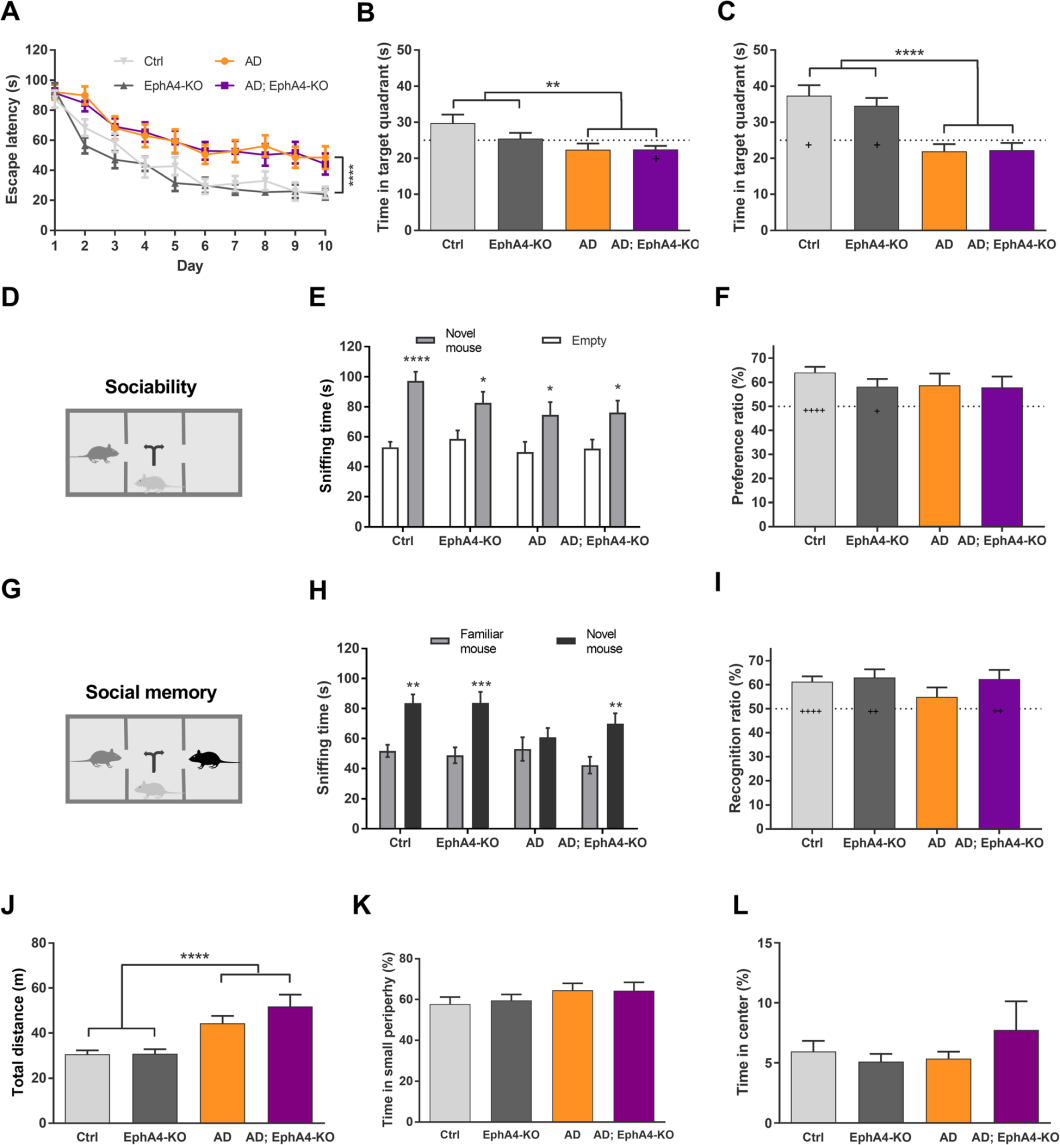
EphA4 loss ameliorates social memory in APPPS1 mice. At 9 months of age, mice were subjected to different cognitive tests to assess memory performance. Escape latency over 10 training days (**a**) and time spent in the target quadrant in probe trial 1 (**b**) and probe trial 2 (**c**) during the MWM test (two-way RM ANOVA and two-way ANOVA with Tukey’s multiple comparison test, and unpaired *t* test to compare to chance level). Sniffing time to the empty cage versus the novel mouse and preference ratio (**d**–**f**) in the sociability trial (unpaired *t* test or Mann-Whitney *U* test and unpaired *t* test compared to chance level, respectively). Sniffing time to the familiar versus the novel mouse and recognition ratio (**g**–**i**) in the social memory trial of the SPSN test (unpaired *t* test or Mann-Whitney *U* test and unpaired *t* test compared to chance level, respectively). Total distance crossed (**j**) and time spent in the small periphery (**k**) and in the center (**l**) of the open field exploration test (two-way ANOVA with Tukey’s multiple comparison test). *N* = 22–28 mice/group. In panels **b**, **c**, and **j**, significant group effects (AD versus non-AD) are indicated as follows: **p ≤ 0.01, ****p ≤ 0.0001. In panels **e** and **h**, significant effects between social subjects (novel mouse versus empty or familiar mouse) are indicated as follows: *p ≤ 0.05, **p ≤ 0.01, ***p ≤ 0.001, ****p ≤ 0.0001. Performance above chance levels (panels **b**, **c**, **f**, and **i**) are indicated as follows: ^+^*p* ≤ 0.05, ^++^*p* ≤ 0.01, ^++++^*p* ≤ 0.0001. If no asterisk or plus sign is shown in the graph, this implies no significance. Abbreviations: MWM Morris water maze, SPSN sociability/preference for social novelty

To determine whether EphA4 knock-down ameliorates the hippocampus-dependent cognitive memory deficits observed in the APPPS1 mouse model, we assessed spatial learning and memory with the Morris water maze test. Ctrl and EphA4-KO mice efficiently learned the location of the hidden platform as reflected in the gradual reduction in time to reach the platform (escape latency) (Fig. 2a). During the probe trials, Ctrl and EphA4-KO mice spent more time in the target quadrant compared to the chance level, showing that they were able to retrieve the information previously learned (Fig. 2b, c). In contrast, AD mice had reduced learning capacities as illustrated by increased time to reach the platform and inability to retrieve information from the previous training sessions in the probe trials (Fig. 2b, c). Loss of EphA4 in AD mice (AD;EphA4-KO) did not affect spatial learning and memory performance in this test (Fig. 2a–c).

We next studied the ability to remember social interactions, another hippocampus-dependent memory function, with the sociability/preference for social novelty (SPSN) test. During the sociability trial, all groups showed normal social behavior as revealed by the preference to explore a novel mouse in comparison with an empty cage (Fig. 2d–f), although this trend did not reach statistical significance for the preference ratio in AD (*p* = 0.085) and AD;EphA4-KO mice (*p* = 0.095). After the introduction of a novel mouse in the social memory trial, Ctrl and EphA4-KO mice preferred to explore the novel mouse in comparison to the familiar mouse. AD mice spent similar time sniffing the novel and the familiar mouse indicative of impaired social memory. This impaired social memory was no longer present in AD;EphA4-KO mice since they showed more interest in the novel mouse (Fig. 2g–i).

As alterations in activity and anxiety levels might affect the performance in the memory tasks, the open field exploration test was used to assess these parameters for the different groups. EphA4-KO mice did not differ from Ctrl mice in activity (total distance covered in the field) and anxiety levels (time spent in the small periphery and center of the field) (Fig. 2j–l). AD mice were more active than Ctrl and EphA4-KO mice with no changes in anxiety (Fig. 2j–l). Loss of EphA4 in AD mice (AD;EphA4-KO) did not affect these parameters (Fig. 2j–l).

### EphA4 knock-down alters hippocampal spine morphology in the proximal stratum radiatum

**Fig. 3:**
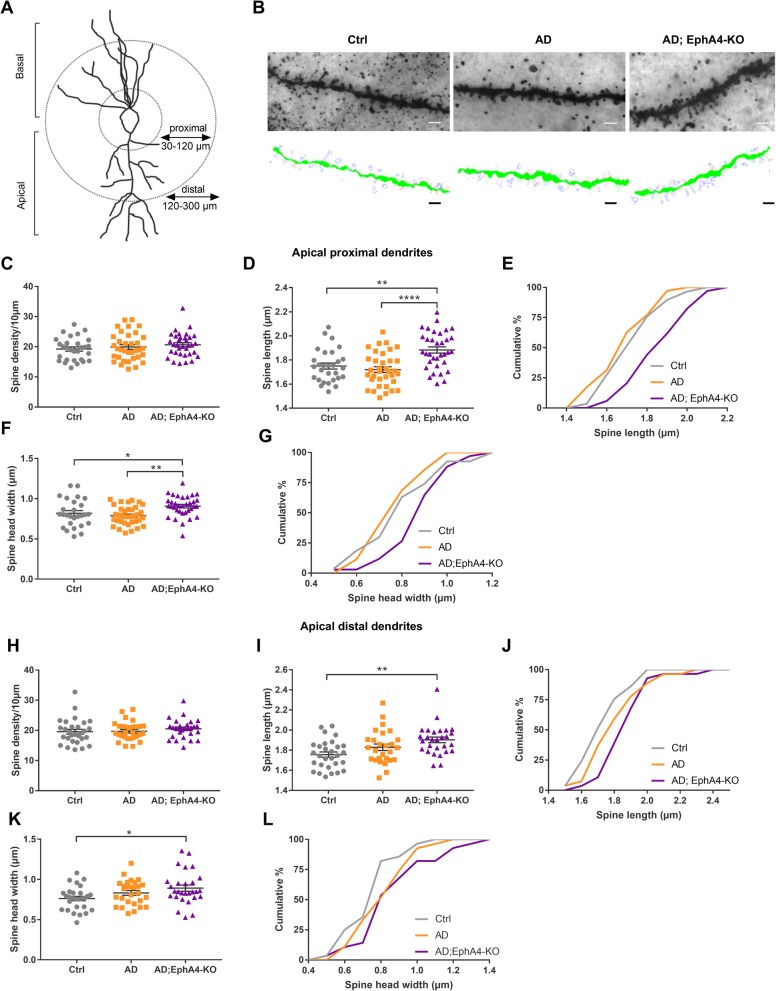
Loss of EphA4 increases dendritic spine length and changes spine morphology in ventral CA1 region. **a** Apical dendritic segments of pyramidal neurons of the ventral CA1 region were randomly chosen and imaged in mice of 10–11 months old. A distinction was made between proximal (30–120 μm from the soma) and distal (120–300 μm from the soma) segments. **b** Representative images and Neurolucida 360 reconstructions of proximal apical dendritic segments of Ctrl, AD, and AD;EphA4-KO mice. Quantifications of spine density (**c**), spine length (**d**, **e**), and spine head width (**f**, **g**) of segments of apical proximal dendrites (one-way ANOVA with Tukey’s multiple comparison test, *N* = 5 mice/group, *n* = 29–35 dendritic segments/group). Quantifications of spine density (**h**), spine length (**i**, **j**), and spine head width (**k**, **l**) of segments of apical distal dendrites (Kruskal-Wallis test with Dunn’s multiple comparison test, *N* = 5 mice/group, *n* = 27–29 dendritic segments/group). *p ≤ 0.05, **p ≤ 0.01, ****p ≤ 0.0001. If no asterisk is shown in the graph, this implies no significance. Scale bar = 2 μm

In order to find out the underlying mechanism responsible for the observed improvement in social memory, we explored alterations in dendritic spine density and/or morphology. Golgi-Cox staining was used to visualize Ctrl, AD, and AD;EphA4-KO mice pyramidal neurons in the ventral CA1 region, an area important for the storage of social memory [30]. Spine density and morphology were measured in dendritic segments derived from apical dendrites in two regions of the stratum radiatum (SR), the proximal SR (30–120 μm from the cell soma) and the distal SR (120–300 μm from the cell soma) [31–33] (Fig. 3a). Spine density and length were similar in AD mice compared to control mice in both proximal and distal apical dendrites (Fig. 3c–e, h–j). EphA4 loss did not alter spine density, but spine length in the proximal SR was longer in AD;EphA4-KO mice versus AD and Ctrl mice (Fig. 3c–e). This increase in spine length in mice with loss of EphA4 was also present in the distal SR when comparing AD;EphA4-KO mice versus Ctrl mice (trending compared to AD mice) (Fig. 3h–j). As increased head width of spines correlates with improved synapse strength, we also measured the head width of the spines [34, 35]. The spine head width did not differ between AD versus control mice in both the proximal and distal SR (Fig. 3f, g, k, l). EphA4 knock-down increased spine head width in AD mice in the proximal SR, while in the distal SR, we only observed this difference in comparison to Ctrl mice (Fig. 3f, g, k, l), similar to the findings on spine length.

### EphA4 knock-down does not alter hippocampal β-amyloid pathology

**Fig. 4:**
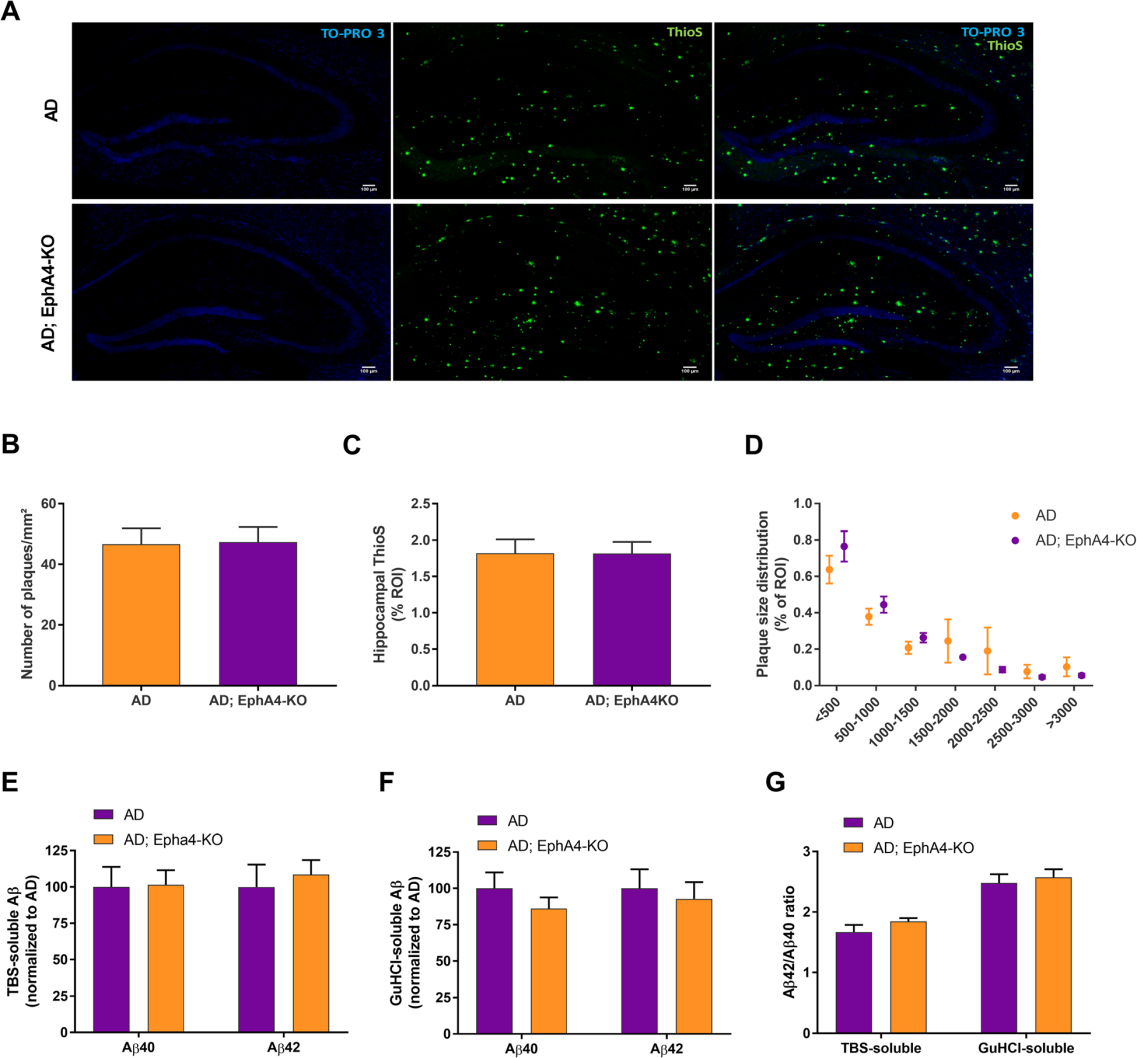
Loss of EphA4 does not alter hippocampal plaque load in APPPS1 mice. **a** Representative images of a Thioflavin S staining to determine hippocampal plaque load in AD and AD;EphA4-KO mice at 10–11 months of age. TO-PRO3 was used to stain cell nuclei. Quantification of the number of plaques/mm^2^ (**b**) and the percentage of the hippocampus positive for ThioS (**c**) in AD versus AD;EphA4-KO mice (unpaired *t* test, *n* = 10–13 mice/group). **d** Quantification of the plaque size distribution (in μm^2^) in the hippocampus of AD and AD;EphA4-KO mice (two-way RM ANOVA, *n* = 10–13 mice/group). Quantification of the levels of TBS-soluble (**e**) and GuHCl-soluble (**f**) Aβ40 and Aβ42 levels (unpaired *t* test, *n* = 11 mice/group). **g** Quantification of the Aβ42/Aβ40 ratio in TBS-soluble and GuHCl-soluble extracts (unpaired *t* test, *n* = 11 mice/group). If no asterisk is shown in the graph, this implies no significance. Scale bar = 100 μm. Abbreviations: ROI region of interest, ThioS Thioflavin S

As APPPS1 mice present with robust β-amyloid pathology [24], we verified whether improved social memory upon EphA4 loss was associated with changes in β-amyloid aggregation. First, plaque density and plaque burden were determined (Fig. 4a–d). Amyloid deposits were highly abundant in AD mice and loss of EphA4 did not modify plaque density or plaque burden. The distributions of plaque sizes were similar between AD and AD;EphA4-KO mice (Fig. 4d). Finally, TBS-soluble and GuHCl-soluble hippocampal Aβ40 and Aβ42 levels were measured. EphA4 knock-down did not change Aβ40 and Aβ42 levels (Fig. 4e, f), nor the Aβ42/Aβ40 ratios (Fig. 4g)*.*

## Discussion

We studied the modifying role of reducing expression of EphA4 in the forebrain on memory function in the APPPS1 mouse model of AD via alterations in spine density and morphology. Our results show that loss of EphA4 improves social memory performance in association with increases in dendritic spine length and head width.

EphA4 knock-down in APPPS1 mice selectively improved social recognition memory, but did not beneficially influence spatial learning and memory. This effect was independent of explorative behavior since activity and anxiety were similar in APPPS1 mice with normal versus reduced EphA4 expression. In constitutive full EphA4-KO mice [36], cognitive deficits are present which is in contrast to our findings in EphA4^flox/flox^ x Camk2aCre mice which revealed no impairments in activity, anxiety, and spatial and social recognition memory. There are several possible explanations for the variation in these results. First, different test paradigms to assess activity, anxiety, and spatial and recognition memory were used. Second, in constitutive EphA4-KO mice, EphA4 is absent during development versus reducing EphA4 levels from the third postnatal week on in EphA4^flox/flox^ x Camk2aCre mice [26] thereby circumventing developmental deficits occurring in constitutive EphA4-KO mice [16]. Third, the mice used in our study have preserved ± 10% of the physiological EphA4 protein levels in the cortex and hippocampus which might be sufficient for normal cognitive function as LTP is not affected in mice with similar EphA4 levels [19].

The specific improvement of social memory, but similar spatial memory performance in APPPS1 mice with EphA4 loss is of interest. One possible explanation for these differences is the differential test sensitivity. Mice rely on the sense of olfaction for social recognition, which is extremely sophisticated in contrast to vision in mice [37, 38]. Hence, we argue that the formation of social recognition memory is an easier task in comparison to the formation of a spatial map based on visual cues. Therefore, effects of loss of EphA4 in the forebrain might be mild and only sufficient to improve social memory, but not spatial memory in the more difficult Morris water maze test. Moreover, various aspects of learning and memory rely on different regions of the brain. In the hippocampus, spatial memory involves preferentially dorsal CA1 and CA3 regions [39–41], while social memory is dependent on ventral CA1 and CA2 activity [30, 42]. In addition, the amygdala has a significant role in the acquisition of social memory [43]. As Cre-recombinase activity in the amygdala of the Camk2aCre mouse was reported to reach similar levels as in the hippocampus and cortex [44], considerable loss of EphA4 levels in this region might contribute to the specific improvement in social memory.

Consecutively, we measured spine density, length, and head width in the ventral CA1 region since the reduction of EphA4 levels was most pronounced in the CA1 region and ventral CA1 activity is indispensable for social memory [30]. Loss of spines is reported to be more pronounced in close proximity to the amyloid plaque in the cortex of APPPS1 mice, as well as in several other mouse models and in patients [45–50]. As a substantial plaque load was present in the stratum radiatum of the ventral CA1, a region in which projections implicated in social memory terminate, we focused spine analysis on this region [42]. Segments from both the proximal and distal stratum radiatum were analyzed as these regions can be selectively affected in AD mouse models [32]. However, we were not able to detect spine loss on apical dendrites in APPPS1 mice, which is in accordance with a previous study [51].

Loss of EphA4 did not increase spine density. Although previous studies reported the normalization of spine numbers in an in vitro AD model upon reduced EphA4 signaling, spine density was not increased upon reduced EphA4 signaling in control conditions with normal spine densities [21, 22]. The absence of spine loss in our AD mouse model hampers the study of the modifying role of EphA4 expression on spine density since this can most likely not increase above normal values. Interestingly, loss of EphA4 induced changes in spine morphology as both spine length and spine head width were increased in the proximal SR. These spine alterations induced by EphA4 loss might underlie social memory improvements in AD mice as increased spine length has been described in individuals with cognitive resilience to AD pathology and is believed to extend the reach of the spines to form new synaptic connections or to connect with degenerating axons [52]. In addition, we assume that the synapses in the proximal SR are stronger in mice with reduced EphA4 levels, as the size of the spine head is directly correlated with synaptic strength [34, 35]. In accordance, a previous study linked reduced EphA4 signaling in non-Aβ conditions to an increased number of mushroom-shaped spines, which are characterized by thin necks, large heads, and the formation of strong and stable synapses [53, 54].

As APPPS1 mice present with robust amyloid pathology, we excluded the involvement of altered amyloid deposition in the improvement in social memory upon EphA4 loss [24]. Although EphA4 is suggested to enhance Aβ generation in vitro [55]*,* amyloid plaque burden and Tris-soluble and GuHCl-soluble Aβ levels did not differ in AD mice with normal versus reduced EphA4 levels.

Although loss of EphA4 is associated with altered spine morphology, further research will need to clarify how these alterations contribute to improved social memory. First, the current work was limited by the inability to measure spine density in close proximity to the beta-amyloid plaques, as the combination of Golgi-Cox staining and plaque visualization was technically not feasible. Novel techniques have recently been developed to combine these techniques and await validation [56]. It would be interesting to examine if spine loss can be detected near beta-amyloid plaques in the APPPS1 mouse model and, when affirmative, if the improvement in social memory is associated with a specific amelioration in plaque-associated spine loss. Second, further investigation of spine subtypes, synapse formation, and synapse electrophysiology could provide more insight in how increased spine length and spine head width underlies the observed improvement in social memory. Last, examination of spine morphology and density in other brain regions such as the amygdala could be of importance to estimate the involvement of other brain regions and to explore possible mechanisms for the specific improvement of social memory upon EphA4 loss, while spatial memory was unaffected.

## Conclusions

Our work demonstrates that loss of EphA4 in the forebrain ameliorates the social memory deficit observed in APPPS1 mice, in association with alterations in spine morphology. We hypothesize that the underlying mechanism of this improvement relates to synaptic function, as changes in spine morphology might be associated with enhanced synaptic strength and connectivity.

## Supplementary information

**Additional file 1: Figure S1**. Protein levels of the human APP and PS1 transgenes remain unaltered by EphA4 loss. Representative images (A,C) and quantifications (B,D) of Western blot analysis with antibodies specific for human APP and PS1 in AD (EphA4 +) and AD;EphA4-KO (EphA4 -) mice (unpaired t-test, *n* = 8–10 mice/group). If no * is shown in the graph, this implies no significance.

## Data Availability

The datasets used and/or analyzed during the current study are available from the corresponding author on reasonable request.

## Abbreviations

AD: Alzheimer’s disease
Aβ: Beta-amyloid
LTP: Long-term potentiation
SCI: Spinal cord injury
hAPP: Human amyloid precursor protein
hPS1: Human presenilin 1
MWM: Morris water maze
SPSN: Sociability/preference for social novelty test
SR: Stratum radiatum
DG: Dentate gyrus
Ctrl: Control
