# Supplementary figures and images for: EphA4 loss improves social memory performance and alters dendritic spine morphology without changes in amyloid pathology in a mouse model of Alzheimer’s disease
Source: Alzheimers Res Ther. 2019 Dec 12;11:102. doi: 10.1186/s13195-019-0554-4 (PMC6909519; doi:10.1186/s13195-019-0554-4)

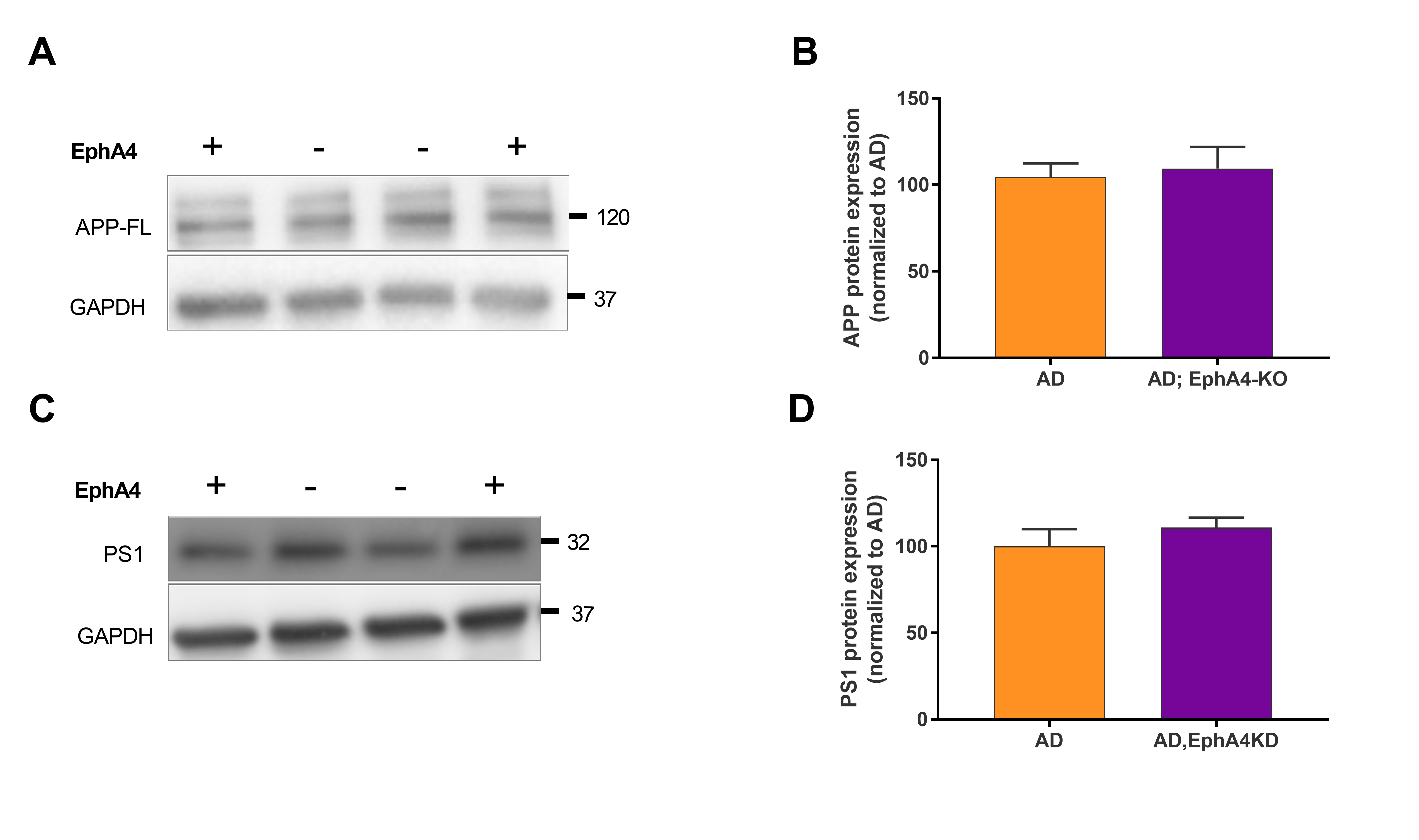

Supplement: Supplementary file 1 — Additional file 1: Figure S1. Protein levels of the human APP and PS1 transgenes remain unaltered by EphA4 loss. Representative images (A,C) and quantifications (B,D) of Western blot analysis with antibodies specific for human APP and PS1 in AD (EphA4 +) and AD;EphA4-KO (EphA4 -) mice (unpaired t-test, n = 8–10 mice/group). If no * is shown in the graph, this implies no significance. [file 13195_2019_554_MOESM1_ESM.tif]
